# “They Were Noble Automatons Who Knew Not What They Did:” Volition in Jaynes' *The Origin of Consciousness in the Breakdown of the Bicameral Mind*

**DOI:** 10.3389/fpsyg.2021.811295

**Published:** 2021-12-20

**Authors:** James W. Moore

**Affiliations:** Department of Psychology, Goldsmiths University of London, London, United Kingdom

**Keywords:** volition, consciousness, bicameral mind, Julian Jaynes, origins, history of psychology

## Abstract

An important question in consciousness research concerns its origins. In Julian Jaynes' book, *The Origin of Consciousness in the Breakdown of the Bicameral* Mind, he suggests that consciousness arose rather recently in human history, sometime between the composition of *The Iliad* and *The Odyssey*. Although Jaynes' work as a theory of consciousness has achieved a great deal of attention (and indeed criticism), what has not been widely noted is the prominent role of volition in his theory. In this article I hope to draw attention to these overlooked aspects of his theory, in particular the fact that volition is central to Jaynes' definition of consciousness and that it is changes in the nature of volitional experience that mark, for Jaynes, the emergence of consciousness.

## Introduction

In Julian Jaynes' book*, The Origins of Consciousness in the Breakdown of the Bicameral Mind* (Jaynes, 1977),^1^ he presented an ambitious theory that turned to the historical record in an attempt to pinpoint the period of human history in which consciousness emerged. For Jaynes, this period occurred sometime between the creation of *The Iliad* and *The Odyssey*, which he suggests were not composed by a single author (i.e., Homer) but rather the result of collective composition over time. According to Jaynes, *The Iliad* was likely composed between 1230BC and 850BC, with *The Odyssey* following *The Iliad* by “at least a century or more” (Jaynes, 1993, p. 272). These two works form the evidential bedrock of this theory, revealing in the content of their narratives the emergence of human consciousness.

Jaynes' theory is generally thought of as one that deals with the origins of consciousness more broadly. However, on closer inspection it is clear that it also has much to say about the origins of human volition. In fact, it could be argued that it is as much a theory of volition as it is of consciousness. One reason for this is that, for Jaynes, self-volition is a defining characteristic of consciousness (see below for further discussion of this). Consequently, the emergence of consciousness manifests itself in changes in the nature of human volition. Although the topic of volition crops up fairly regularly throughout Jaynes' book, I will focus primarily on what he has to ^1^ say about it in the context of *The Iliad* and *The Odyssey* (it should be noted here that the range of evidence Jaynes presents is far broader than these two classic works, encompassing a variety of historical sources and cultures, as well as contemporary evidence, such as the symptoms of schizophrenia and the phenomenon of hypnosis).

Anyone familiar with Jaynes theory is likely to be aware of the mixed critical reception of Jaynes' theory. This criticism is important to acknowledge and is something I address towards the end of this article. However, whilst important, my concern here is not the veracity of Jaynes' theory. Instead, my aim is to draw attention to those (overlooked) features of Jaynes' theory that directly relate to human volition.

## The Theory

There are three elements to Jaynes' theory of the origins of consciousness: (1) his definition of consciousness, (2) his notion of the bicameral mind, and (3) his argument that consciousness emerged following the breakdown of the bicameral mind. In terms of his definition of consciousness, Jaynes starts by telling us what consciousness is *not*, chipping away at the common misconceptions that he believes have hindered a more complete understanding of this phenomenon. Following this process of elimination, Jaynes arrives at the following definition of consciousness:

Subjective conscious mind is an analogue of what is called the real world. It is built up with a vocabulary or lexical field whose terms are all metaphors or analogues of behaviour in the physical world. Its reality is of the same order as mathematics. It allows us to shortcut behavioural processes and arrive at more adequate decisions. Like mathematics, it is an operator rather than a thing or repository. And it is intimately bound up with volition and decision (Jaynes, 1993, p. 55).

On this view, he sees language as fundamental to consciousness, which then opens up the possibility of unlocking the origins of consciousness by studying our linguistic historical record; an endeavour that Dennett (1986) calls “software archaeology.” It is interesting to note here that Jaynes' definition of consciousness, quoted above, is also relevant to our discussion of volition, given that he explicitly links consciousness and volition.

With the definition of consciousness mapped out, we proceed to Jaynes' idea of the bicameral mind. According to Jaynes, prior to the emergence of consciousness, the human mind was bicameral i.e., it was split into two parts: a decision-making part and a follower part. Importantly, neither one of these separate parts was conscious. For simple actions, bicameral people were creatures of habit, following well-established routines and patterns of behaviour. Every so often, however, a situation would arise for which routines and habits were not sufficient. In these situations the decision-making part of the mind was recruited. This would direct behaviour by issuing an auditory command. Crucially, these commands were not regarded as self-generated. Instead, bicameral people experienced them as being issued by an external agent. For Jaynes, this property of the bicameral mind explains the origin of gods in human societies—humans regarded these auditory hallucinations as the words of their god(s).

This aspect of the bicameral mind, the outsourcing of volition and decision making to putatively external agents (gods), is directly relevant for our discussion of volition in the context of Jaynes' theory. For Jaynes, the absence of consciousness is actually marked by an absence of self-volition. Bicameral people did not feel they were responsible for their decisions and actions, and this is because they were not conscious. The apparent causal agents in human affairs were not humans but gods.

According to Jaynes, the bicameral mind was an adaptation to the emergence of agricultural societies. This wrought seismic social changes as humans shifted away from living within small hunter-gatherer groups, and instead started to lay down roots and to trade. One of the most profound changes to result from this process was the rapid expansion of the population.

One issue to arise from this population expansion was of maintaining social control. This was easily managed and policed in small hunter-gatherer societies, where leaders were a physical presence. However, in these new larger societies, social control was not so easy, as humans were physically distanced from their rulers. According to Jaynes, the bicameral mind emerged as a solution to this problem. The controlling influence of leaders and gods could be maintained in the form of auditory hallucinations emanating from the decision-making chamber within each individual's own psyche. These auditory hallucinations “became the way of controlling larger groups” (Jaynes, 1986, p. 10).

For Jaynes, the bicameral mind was a fragile solution to this budding social complexity, and it only existed for about 7,000 years (emerging around 9,000BC). The success of these agricultural societies led to further population growth, which in turn made the job of social control harder to sustain, even with the hallucinated words of gods directing the populous' behaviour. On top of this, agricultural societies went through further cultural and intellectual shifts that served to undermine the efficacy of the bicameral mind. One change in particular posed a problem: the development of writing. According to Jaynes, writing allowed humans to escape the tyranny of their auditory hallucinations. Once something is written down, such as a law or code of conduct, one can walk away from it and return to it. In this way the auditory hallucinations lost their power and influence, which was instead transferred to the written word.

At this point in human history, humans started to lose their gods. Jaynes quotes from the *Ludlul Bel Nemeqi*—a Mesopotamian poem written during the time of the bicameral mind's breakdown—which clearly expresses this loss:

My god has forsaken me and disappeared,

My goddess has failed me and keeps at a distance,

The good angel who walked beside me has departed.

To explain this abandonment, humans invented heavens and underworlds—places to which their deities retreated. And in the absence of these gods, a new psyche emerged, one that we too would now recognise. This is the conscious mind that we are familiar with, where decisions and actions are issued from within. Jaynes believed that it was at this point in human history that consciousness emerged. Contrasting *The Odyssey* with the earlier *Iliad*, Jaynes writes:

the bicameral mind by its very definition directs much less of the action. The gods have less to do, and like receding ghosts talk more to each other—and that so tediously! The initiatives move from them, even against them, towards the work of the more conscious human characters (Jaynes, 1993, p. 273).

So it is the emergence of conscious *will* that signals the emergence of consciousness. As I have already pointed out, for Jaynes, consciousness entails self-volition, as well as decision-making. It is also the emergence of this volitional aspect of human mental life that marks the emergence of human consciousness, and which can be seen in the written historical record.

## The Iliad, The Odyssey and The Emergence of Self-Agency

According to Jaynes, the shift from god-directed automata to self-determined agents can be seen in certain fundamental narrative differences between *The Iliad* and *The Odyssey*. He first points out that our modern concept of the will is entirely absent from *The Iliad* (the older of the two texts), noting that “there is... no concept of will or word for it, the concept developing curiously late in Greek thought. Thus, Iliadic men have no will of their own and certainly no notion of free will” (Jaynes, 1993, p. 70). At another point in his book, Jaynes describes the soldiers of the Trojan war as being “...not at all like us. They were noble automatons who knew not what they did” (Jaynes, 1993, p. 75). Here is the startling claim that up to this very recent point in human history, there was no experience of self-volition. Human will was “outsourced” to the gods.

So what form did the gods take and what was their nature? An important clue comes from the fact that in *The Iliad*, gods were very much a part of the natural order of things. They were not in possession of super-natural powers or qualities *per se*. Instead they seemed rather akin to the humans whose minds were their creators. Jaynes talks about the “amazement or wonder” (Jaynes, 1993, p. 74) that is sometimes evoked by an interaction with a god, but he also points out that this is something akin to the feeling we get when a solution to a difficult problem suddenly appears to us. It is an extraordinary feeling, but it in no way feels super-natural. So the god-driven agency experienced by bicameral people was not out of this world, but was consistent with the natural order of things and the rules and limitations that govern human behaviour. This makes sense given that the gods themselves were human-made.

Jaynes also speculates about the neural architecture of the bicameral mind, and the way in which this shaped the experience of volition. Simply put, the bicameral mind can be mapped on to the two-hemispheres of the brain. The right hemisphere was the controller, storing up commands that could be issued when needed. These commands were sent to the left hemisphere *via* the anterior commissure. This information transfer corresponds, at the phenomenological level, to the experienced commands from gods. The anterior commissure, the small link between hemispheres, is the conduit of volition in the bicameral mind.

It is important here to draw attention to the issue of responsibility. The great psychological and social weight of self-agency comes from the fact that one can be held responsible for one's actions. What then of bicameral people and the societies in which they lived? Jaynes does not dwell on this issue, but is unequivocal in his stance, making the rather bold claim that “...early civilizations had a profoundly different mentality from our own, that in fact men and women were not conscious as are we, were not responsible for their actions, and therefore cannot be given the credit or blame for anything that was done over these vast millennia of time” (Jaynes, 1993, p. 201). Although perfectly consistent with his theory, Jaynes' suggestion that the notion of responsibility was absent from human societies until very recently is rather jarring. If true (and of course, with Jaynes' theory, this is not a given) it would force us to re-write the very narrative of human history.

So the experience of self-agency was absent during the bicameral era. The subsequent breakdown of the bicameral mind that followed the era of *The Iliad* therefore created something of a volitional void—humans no longer felt commanded by their gods. According to Jaynes, before this void was filled with a modern self-agency, there was an intermediary solution in the form of divination. Humans turned away from gods and towards surrogate decision-making systems located in the external world. The aim of divination was to summon up the commands of these lost gods, and could be achieved through a variety of means, such as the casting of lots. Divination rituals acted as external proxies for the hallucinated commands that had previously controlled action in bicameral people.

Jaynes notes that the nature of divination changed over time and that the favoured practises seemed to ever more closely reflect the modern structure of consciousness, with humans edging closer towards a full experience of self-agency. This process ultimately brings us to *The Odyssey*, and it is in the narrative of *The Odyssey* that, according to Jaynes, we can see this proto-agency becoming a more fully-fledged self-agency. For example, the role of the gods has shifted, from centre-stage protagonists to bit part players in the theatre of human agency. We also see the more prominent role of divination, with frequent referencing of seers and omens.

As well as changes in the content of the narrative, the language itself also seems to change. Jaynes notes that, compared with *The Iliad*, in *The Odyssey* there are changes in the frequency of certain key words. For example, according to Jaynes there is an increase in the frequency of the word *noos* in *The Odyssey*, which Jaynes defines as the conscious mind. Just as importantly, it is the *noos* that now directs much more of the action in this work compared with its predecessor, reflecting a shift from god-driven to self-driven agency.

Jaynes also suggests that *The Odyssey* itself can be read as a metaphor for this shift in human mentality, that the narrative is one of self-discovery and the emergence of new and changed identities. Jaynes' description of this is worth quoting here at length:

And as this series of storeys sweeps from its lost hero sobbing on an alien shore in bicameral thrall to his beautiful goddess Calypso, winding through its world of demigods, testings, and deceits, to his defiant war whoops in a rival-routed home, from trance through disguise to recognition, from sea to land, east to west, defeat to prerogative, the whole long song is an odyssey towards subjective identity and its triumphant acknowledgment out of the hallucinatory enslavements of the past. From a will-less gigolo of a divinity to the gore-spattered lion on his own hearth, Odysseus becomes “Odysseus” (Jaynes, 1993, p. 277).

So we see reflected in the narrative of *The Odyssey* and the journey of Odysseus, the human journey towards consciousness. Interestingly, Jaynes explicitly notes that this journey involves a shift in the nature of human agency from the “will-less gigolo of a divinity” to a more fully-fledged agent. So again, Jaynes here is equating, at least to some extent, the emergence of consciousness with the emergence self-agency.

## Conclusion

From this brief overview I hope to have drawn attention to the importance of volition for Jaynes' theory of consciousness. In my analysis two themes have emerged: the first is that the experience of self-volition appeared fairly recently in human history, and the second is that, for Jaynes, it was the appearance of self-volition that signalled the emergence of consciousness following the breakdown of the bicameral mind. The latter is perhaps the most intriguing feature of Jaynes' theory from a contemporary theoretical perspective. This is because it implies that self-volition is a defining feature of human consciousness, perhaps even *the* defining feature. On this view, volition may be foundational to consciousness—only once an organism has internalised the causes its behaviour (thus linking them to the “self”) can that organism be said to be conscious.

Before concluding, it is important briefly to acknowledge the theory's critical reception, especially within academic circles (for a more extensive review see, for example Rowe, 2012). A number have found fault with various aspects of Jaynes' theory. For example, in an early review Block (1977) argues that if, on a superficial level, the narrative of ancient texts implies a profound difference in mentality, the most plausible interpretation of this is not that the mentality was profoundly different, but that the ancients' interpretation of their internal lives was different to our own. That is, the most likely scenario, according to Block, is that they had minds like ours, but a very different *theory* of mind.

It has also been noted that Jaynes' theory relies heavily on evidence from the Near and Middle East (Carr, 2006; Rowe, 2012). This is because, according to Rowe (2012), this is where the record is strongest. Inevitably this exposes Jaynes to the criticism that his theory is culturally biassed. Although it would be wrong to accuse Jaynes of an exclusive focus on these cultures (for example, he spends time in his book looking at evidence from Mesoamerican cultures such as the Maya), the fact remains that the theory relies heavily on a culturally limited evidence-base. The extent to which his theory may or may not apply to other cultures is an open question, and one that more recent scholars have started to consider (see, for example Carr, 2006, and his application of Jaynes' theory to ancient Chinese ancestral sacrifices).

Despite this criticism, there have also been notable defenders of Jaynes' theory, or at least certain aspects of his theory. For example, Dennett (1986) defends what he calls Jaynes' “top-down” approach to the problem of consciousness. Dennett is also sympathetic to Jaynes' ideas on the emergence of consciousness, namely that it may have happened relatively recently and that social/environmental factors were the driving force behind its emergence.

Although it is important to note the critical reception of Jaynes' theory, my aim in this article has not been to establish the veracity of that theory in terms of the origins of consciousness and human volition. Instead, my aim in this article is a modest one—to offer the reader a primer on the prominence of volition in Jaynes' theory. In doing so, I hope to have shed new light on what remains an important (if flawed) contribution to the field of consciousness research.

## Author Contributions

The author confirms being the sole contributor of this work and has approved it for publication.

## Conflict of Interest

The author declares that the research was conducted in the absence of any commercial or financial relationships that could be construed as a potential conflict of interest.

## Publisher's Note

All claims expressed in this article are solely those of the authors and do not necessarily represent those of their affiliated organizations, or those of the publisher, the editors and the reviewers. Any product that may be evaluated in this article, or claim that may be made by its manufacturer, is not guaranteed or endorsed by the publisher.

## Footnotes

^1^Jaynes' book was first published in 1977. However, the version of the book that I will be drawing quotations from is a later edition: Jaynes (1993).
